# Dirty Electricity Elevates Blood Sugar Among Electrically Sensitive Diabetics and May Explain Brittle Diabetes

**DOI:** 10.1080/15368370802072075

**Published:** 2008-06-06

**Authors:** Magda Havas

**Affiliations:** Environmental & Resource Studies, Trent University, Peterborough, Ontario, Canada

**Keywords:** Radio frequency, Transients, Dirty electricity, Power quality, Plasma glucose, Blood sugar, Insulin, GS filters, Electrohypersensitivity, Brittle diabetes, Type 3 diabetes, Type 2 diabetes, Type 1 diabetes

## Abstract

Transient electromagnetic fields (dirty electricity), in the kilohertz range on electrical wiring, may be contributing to elevated blood sugar levels among diabetics and prediabetics. By closely following plasma glucose levels in four Type 1 and Type 2 diabetics, we find that they responded directly to the amount of dirty electricity in their environment. In an electromagnetically clean environment, Type 1 diabetics require less insulin and Type 2 diabetics have lower levels of plasma glucose. Dirty electricity, generated by electronic equipment and wireless devices, is ubiquitous in the environment. Exercise on a treadmill, which produces dirty electricity, increases plasma glucose. These findings may explain why brittle diabetics have difficulty regulating blood sugar. Based on estimates of people who suffer from symptoms of electrical hypersensitivity (3–35%), as many as 5–60 million diabetics worldwide may be affected. Exposure to electromagnetic pollution in its various forms may account for higher plasma glucose levels and may contribute to the misdiagnosis of diabetes. Reducing exposure to electromagnetic pollution by avoidance or with specially designed GS filters may enable some diabetics to better regulate their blood sugar with less medication and borderline or pre-diabetics to remain non diabetic longer.

## Introduction

*Diabetes mellitus* is increasing globally. According to the World Health Organization, in 1985 the global population of diabetics was 30 million (0.6% of the world population). This increased to 171 million (2.8% of the global population) by 2000, and it is expected to more than double to 366 million (4.5% of the global population) by 2030 (Wild et al., 2004; U.S. Census Bureau, 2005). Doctors attribute this rise in diabetes to poor diet and limited exercise, resulting in obesity, and seldom look for causes other than lifestyle and genetics.

This article presents a paradigm shift in the way we think about diabetes. In addition to Type 1 diabetics, who produce insufficient insulin, and Type 2 diabetics, who are unable to effectively use the insulin they produce, a third type of diabetes may be environmentally exacerbated or induced by exposure to electromagnetic frequencies.

Our increasing reliance on electronic devices and wireless technology is contributing to an unprecedented increase in our exposure to a broad range of electromagnetic frequencies, in urban and rural environments and in both developed and developing countries. This energy is generated within the home by computers, plasma televisions, energy efficient lighting and appliances, dimmer switches, cordless phones, and wireless routers, and it can enter the home and work environment from nearby cell phone and broadcast antennas as well as through ground current.

Although the position of most international health authorities, including the World Health Organization, is that this form of energy is benign as long as levels remain below guidelines, an increasing number of scientific studies report biological and health effects associated with electromagnetic pollution well below these guidelines (Sage and Carpenter, 2007). Epidemiological studies have documented increased risks for childhood leukemia associated with residential magnetic fields exposure (Ahlbom et al., 2000), greater risk for various cancers with occupational exposure to low-frequency electric and magnetic fields (Havas, 2000), miscarriages (Li et al., 2002), Lou Gehrig’s disease (Neutra et al., 2002), brain tumors associated with cell phone use (Kundi et al., 2004), as well as cancers and symptoms of electrical hypersensitivity (EHS) for people living near cell phone and broadcast antennas (Altpeter et al., 1995; Michelozzi et al., 2002). Laboratory studies report increased proliferation of human breast cancer cells (Liburdy et al., 1993), single- and double-strand DNA breaks (Lai and Singh, 2005), increased permeability of the blood brain barrier (Royal Society of Canada, 1999), changes in calcium flux (Blackman et al., 1985), and changes in ornithine decarboxylase activity (Salford et al., 1994).

In this article, changes in plasma glucose, in response to electromagnetic pollution, for numerous measurements on four subjects—two with Type 1 diabetes taking insulin and two non medicated with Type 2 diabetes—are described. They include men and women, ranging in age from 12–80, as well as individuals recently diagnosed and those living with the disease for decades.

### Case 1: 51-Year Old Male with Type 2 Diabetes

**Figure 1:**
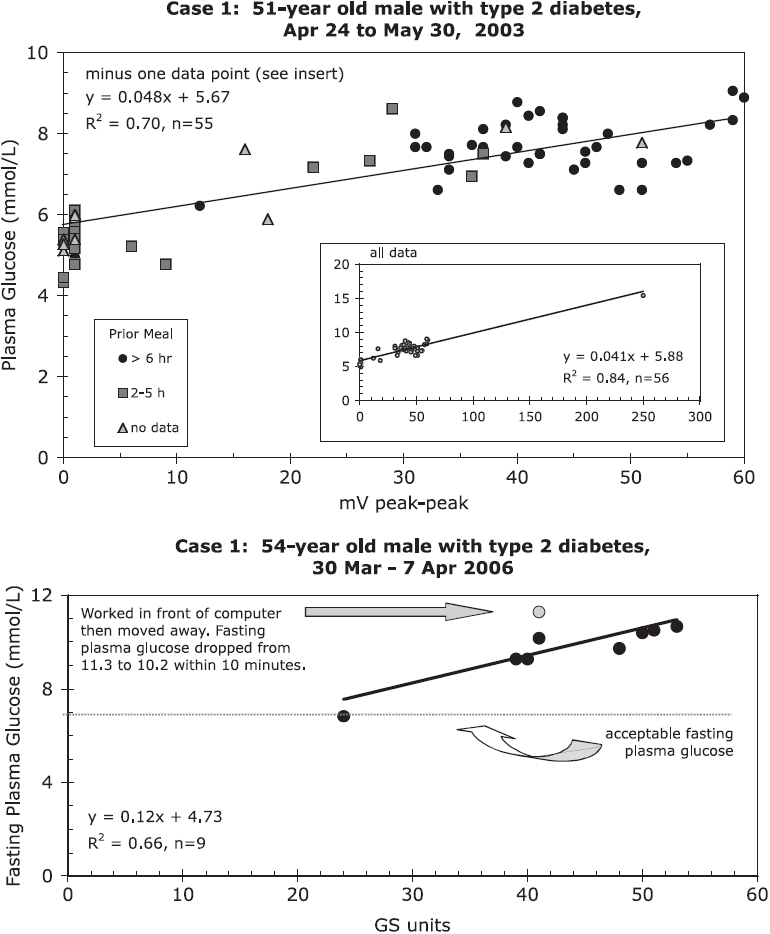
Case 1: *Upper chart*: Plasma glucose levels of a 51-year old male with Type 2 diabetes exposed to different levels of power quality. Insert shows the entire data set with one very high plasma glucose reading that was recorded during a period of high exposure to dirty electricity. *Lower chart*: Three years later, fasting plasma glucose levels correspond to power quality measured in GS units. Time spent in front of computer resulted in higher plasma glucose levels that dropped 1.1 mmol/L [19.8 mg/dL] 10 min after moving away from computer. Note that we have scaled both plots the say way in Fig. 1.

A 51-year old male with Type 2 diabetes, taking no medication, monitored his plasma glucose levels from April 24 to May 30, 2003. He also monitored the dirty electricity in his home using a Protek 506 Digital Multimeter connected to a ubiquitous filter (Graham, 2000) to remove the 60-Hz signal and its harmonics. Measurements were taken in the morning and randomly throughout the day. Low or no readings of dirty electricity were taken in an electromagnetic clean environment far from power lines and cell phone antennas (Fig. 1 upper graph). Three years later, the microsurge meter became available and Case 1 monitored his blood sugar levels once more (Fig. 1 lower graph). This meter provides a digital readout of the absolute changing voltage as a function of time (|dv/dt|, expressed as GS units) for the frequency range 4–100 kHz and with an accuracy of ±5% (Graham, 2003).

Figure 1 shows a positive correlation between dirty electricity and plasma glucose levels taken randomly during the day (upper graph) and first thing in the morning (lower graph). His elevated plasma glucose is unrelated to eating. Working on a computer increases blood sugar, but these values decrease as much as 0.11 mmol/L* [2 mg/dL] per minute after moving away from the computer. Blood viscosity decreased as his plasma glucose levels dropped.

Case 1 also documented rapid changes in blood sugar as he moved from a medical clinic (environment with dirty electricity), to his parked vehicle (no dirty electricity), and back to the medical clinic. His blood sugar levels changed significantly within 20 min. His endocrinologist classified him as *pre-diabetic* when his blood sugar was tested immediately upon entering the medical clinic and as a *Type 2 diabetic* after a 20-min wait in the medical clinic. Measurement of blood sugar needs to be done in an electromagnetically clean environment to prevent misdiagnosis and to accurately determine the severity of the disease.

### Case 2: 57-Year Old Female with Type 2 Diabetes

**Figure 2:**
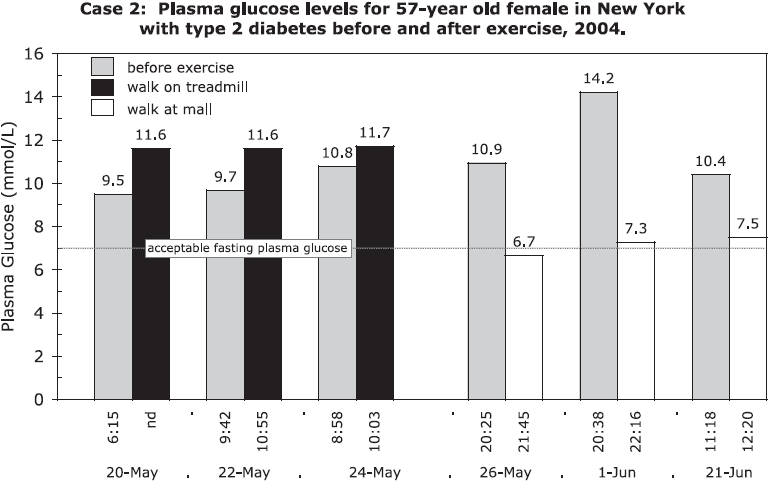
Case 2: Plasma glucose levels for a 57-year old female in New York with Type 2 diabetes, before and after walking for 20–30 min on a treadmill in her home and after hours at a mall.

A 57-year old female with Type 2 diabetes takes no medication and controls her plasma glucose with exercise and a hypoglycemic diet. When she exercised by walking for 20–30 min at a mall after hours, her blood sugar levels dropped from a mean of 11.8 to 7.2 mmol/L [212 to 130 mg/dL] (*p* = 0.045). When she walked on a treadmill, her blood sugar levels increased from 10 to 11.7 mmol/L [180 to 211 mg/dL] (*p* = 0.058) (Fig. 2). Treadmills have variable speed motors and produce dirty electricity.

Doctors recommend exercise for patients with diabetes. However, if that exercise is done in an electromagnetically dirty environment, and if the patient is sensitive to this form of energy, it may increase stress on the body and elevate levels of plasma glucose, as in Case 2.

This subject also measured her plasma glucose as she moved from an environment with dirty electricity to one that was clean, and back again. Her blood sugar in the dirty environment was 12.5 mmol/L [225 mg/dL] and within 20 min in the clean environment dropped to 10.6 mmol/L [191 mg/dL]. Within 5 min after returning to the dirty environment, her blood sugar rose to 10.8 mmol/L [194 mg/dL] and 15 min later to 12.6 mmol/L [227 mg/dL]. She did not eat or exercise during this period. Her elevated plasma glucose levels were associated with headaches, nausea, and joint pain in her home, where she was exposed to both dirty electricity and radio frequency radiation from nearby cell phone antennas. These exposures and symptoms were absent in the clean environment.

### Case 3: 80-Year Old Female with Type 1 Diabetes

An 80-year old female with Type 1 diabetes, who takes insulin (Humlin® 70/30) twice daily, documented her blood sugar levels before breakfast and before dinner for one week. On June 12, 2004, the dirty electricity in her home was reduced from an average of 1,550 GS units (range: 600 to > 2,000) to 13 GS units (range 11 to 22) with Graham/Stetzer filters (GS filters). These filters provide a short to high frequency, and, thus, reduce transients on electrical wiring with an optimal filtering capacity between 4 and 100 kHz (Graham, 2000, 2002, 2003). They are similar to capacitors installed by industry to protect sensitive electronic equipment from power surges and to adjust the power factor. GS units measure the energy associated with dirty electricity (amplitude and frequency) and are a function of changing voltage with time (dv/dt). Dirty electricity can be measured using an oscilloscope or multi-meter set for peak-to-peak voltage or a Microsurge meter that provides a digital readout (GS units) and is easily used by non professionals.

Case 3 had mean fasting plasma glucose of 9.5 mmol/L [171 mg/dL] without the GS filters and 6.6 mmol/L [119 mg/dL] with the GS filters (*p* = 0.02) (Table 1). Her evening blood sugar did not change appreciable during this period, although it did differ on days she was away from home. She was able to more than halve her insulin intake (*p* = 0.03) once the GS filters reduced the dirty electricity in her home (Table 1).

**Table 1:** Case 3: Plasma glucose levels and daily insulin injections (Humulin® 70/30) for an 80-year old woman with Type 1 diabetes before and while GS filters were installed in her home in Arizona

	Plasma Glucose (mg/dL)		
		
Date 2004	Morning (7 am)	Evening (5 pm)	Daily Insulin (units)
Without GS Filters: Dirty Electricity 1,550 GS units			
June 5	158	239•	56
June 6	158	167	56
June 7	160	113•	56
June 8	180	104	0
June 9	180	144	56
June 10	151	76	56
June 11	116	229	28
Mean (sd)	171 (20)	153 (63)	44 (22)
With GS Filters: Dirty Electricity 13 GS units (installed June 12)			
June 13	86	194	0
June 14	140	94	25
June 15	115	178	0
June 16	112	135	15
June 17	131	175	20
June 18	167	250•	50
June 19	70	169	22
June 20	133	126	22
Mean (sd)	119 (31)	166 (49)	19 (16)
2-tailed *t*-test	*p* = 0.002**	*p* = 0.69	*p* = 0.03*

• Subject was away from home during the day.

### Case 4: 12-Year Old Male with Type 1 Diabetes

A mother and her 5 children, who were all home schooled, began to develop intermittent, excruciating headaches during the fall of 2002 in rural Wisconsin, shortly after they had a new septic system installed. The headaches continued and a power quality expert measured high levels of dirty electricity and ground current, possibly attributable to the septic system installation.

In December 2002, one child, a 12-year old male, was hospitalized and diagnosed with Type 1 diabetes. His younger sister had been living with diabetes since the age of 3 months and was one of the youngest children diagnosed with diabetes in the United States.

**Figure 3:**
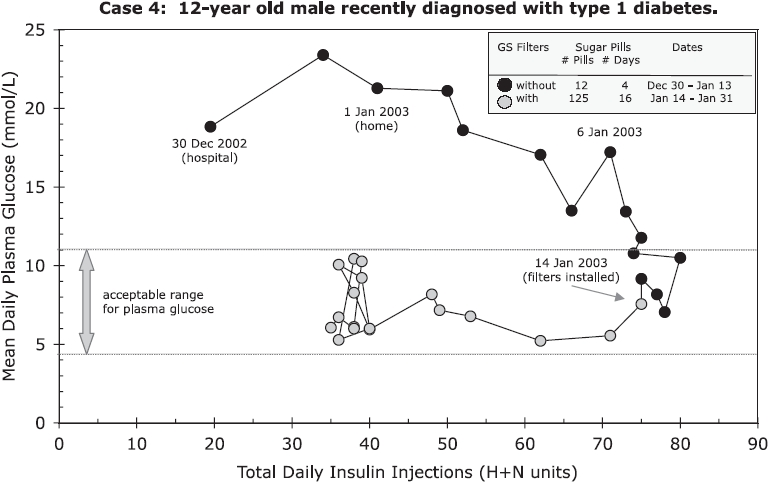
Case 4: Sequence of mean daily plasma glucose levels and total daily insulin injections for 12-year old male with Type 1 diabetes who was admitted to hospital in December 2002 and returned home on January 1, 2003. On January 14, 2003, GS filters were installed in his home to improve power quality.

On January 14, 2003, the family installed GS filters to help alleviate their symptoms of electrical hypersensitivity. The headaches disappeared and the family’s health began to improve. Shortly after the GS filters were installed, the mother had great difficulty controlling her son’s blood sugar. She couldn’t reduce the amount of insulin fast enough to keep it within an acceptable range and needed to give him sugar pills to prevent hypoglycemia (Fig. 3). He was taking a combination of Humalog® (H-insulin, a short-acting insulin) and Humulin® NRT (N-insulin, a long-lasting insulin).^1^ During this period, her daughter’s blood sugar levels began to drop as well.

Doctors attribute the short-term improvement in blood sugar to the ‘‘honeymoon period’’, which is observed among some diabetics shortly after diagnosis and lasts from weeks to months and occasionally for years (Bernstein, 2003). The honeymoon period cannot explain the response of the subject’s sister, who had been living with Type 1 diabetes for years, and who also had lower plasma glucose levels and difficulty regulating her insulin within an acceptable range after the GS filters were installed and the dirty electricity was reduced.

**Figure 4:**
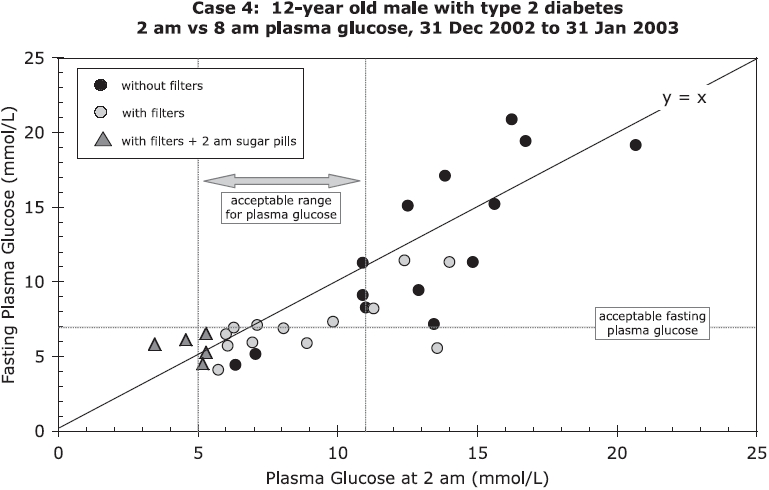
Case 4: Fasting (8 am) and 2 am plasma glucose levels for 12-year old male with Type 1 diabetes with and without GS filters. NOTE: Sugar pills were administered at 2 am for 5 d to prevent hypoglycemia while filters were installed.

Case 4 had higher levels of plasma glucose at 8 am (fasting) than at 2 am on some days before the GS filters were installed. This was not observed with the filters, except when sugar pills were taken at 2 am to deliberately increase blood sugar (Fig. 4). In Wisconsin, dirty electricity often increases in the middle of the night, beginning at 2–3 am and lasting from minutes to hours, as the electric utility makes changes in its system.

## Discussion

These results show that plasma glucose levels, in the Type 1 and Type 2 diabetic cases reported, respond to electromagnetic pollution in the form of radio frequencies in the kHz range associated with indoor wiring (dirty electricity). Type 1 diabetics require less insulin in an electromagnetically clean environment and blood sugar levels for Type 2 diabetics increase with increasing exposure to dirty electricity.

In May 2006, a long-term health care facility in Ontario, Canada installed GS filters to reduce dirty electricity. Of the five diabetic residents, for whom data were available, two (aged 87 and 88) were insulin-dependent Type 1 diabetics. Both had significantly lower fasting plasma glucose levels (*p* < 0.01) after the GS filters were installed. Their insulin intake did not change during this period and nursing staff had to give them orange juice on several occasions to prevent hypoglycemia. The levels of plasma glucose of the remaining three, who were Type 2 diabetics, did not change during this period.

The GS filters, used in this study have been tested at the Yoyogi Natural Clinic in Japan (Sogabe, 2006). Three people participated in the study. Three hours after eating, their blood sugar was 6.3, 7.7, 17.9 mmol/L [113, 139, and 322 mg/dL] in an environment with more than 2,000 GS units of dirty electricity. GS filters reduced the dirty electricity to 30–35 GS units and, within 30 min, their plasma became less viscous and their blood sugar dropped to 5.6, 6.1, 16.1 mmol/L [101, 110, 290 mg/dL], respectively.

The person with the highest plasma glucose levels was a 28-year old male with Type 2 diabetes and fasting plasma glucose levels of 16.7 mmol/L [300 mg/dL]. Despite taking 250 mg of Glycoran®, 3 times a day, and 12 mg of Amaryl®, spread throughout the day, he still had difficultly regulating his blood sugar. Three days after installing 4 GS filters in his home, his blood sugar dropped to 6.9 mmol/L [124 mg/dL] and he was feeling well. He had been unable to achieve such low values with medication alone.

In this study, we classify diabetics whose blood sugar responds to electromagnetic pollution as Type 3 diabetics. In contrast to true Type 1 diabetics who produce insufficient insulin and true Type 2 diabetics who are unable to effectively use the insulin they produce, Type 3 diabetics are responding to environmental triggers that affect blood sugar readings and blood viscosity. These individuals may be better able to regulate plasma glucose by controlling their exposure to frequencies in the low RF range, and thus differ from true Type 1 and Type 2 diabetics whose blood sugar is not affected by this type of electromagnetic exposure.

The increase in blood viscosity with increasing exposure to dirty electricity is a critical observation. If this turns out to be the case among electrosensitive individuals, it may explain the symptoms of headaches, chest pain, higher blood pressure, blurred vision, and fatigue.

The percentage of diabetics who are likely to be affected by electromagnetic energy is unknown, but if the values are similar to those suffering from symptoms of electromagnetic hypersensitivity (EHS), 3–35% of the population (Philips and Philips, 2006), then globally between 5 and 60 million existing diabetics may have Type 3 diabetes as described in this study.

There is a growing body of *in vivo, in vitro,* and epidemiological evidence, which suggests a relationship between plasma glucose levels, insulin secretion, and exposure to electromagnetic energy at frequencies both lower and higher than the ones we tested in this study.

Altpeter et al. (1995) reported that for people living within a 2 km radius of a short-wave transmitter, in Schwarzenburg, Switzerland, the odds ratio (OR) for diabetes was 1.93 when compared with a population further away. There was a significant linear correlation (R^2^ = 0.99) between daily median RF exposure and incidence of diabetes. The highest RF readings, recorded in the nearest zone (51 mA/m), were well below the International Radiation Protection Agency’s 1988 guidelines of 73 mA/m. Those living near the transmitter also had difficulty falling and staying asleep, were restless, experienced weakness and fatigue, and had both limb and joint pain with statistically significant odds ratios between 2.5 and 3.5. These symptoms are typical of radio wave sickness or electrical hypersensitivity (Firstenberg, 2001). Failure of the transmitter for a 3-d period was associated with improved sleep and, hence, these reactions are biological not psychological.

Beale et al. (2001) reported that the prevalence of chronic illness, asthma, and Type 2 diabetes was linearly related to 50-Hz magnetic field exposure for adults living near transmission lines. For Type 2 diabetes, the crude OR was 8.3 (95% CI 1 to 177), but the OR adjusted for possible confounders (age and ethnicity) was reduced to 6.5 and was not statistically significant (*p* > 0.05). Epidemiological studies of power lines tend to focus on cancers, rather than diabetes, and, hence, limited information of this type is available.

Litovitz et al. (1994) exposed diabetic subjects to 60-Hz magnetic fields between 0.2–1 μT (2–10 mG) and noticed that blood glucose levels increased above 0.6 mT. No statistical tests were reported and no attempt was made to measure frequencies other than 60 Hz. Magnetic flux densities above 0.6 μT are realistic near transmission lines and overlap with the range documented in the Beale study (2001).

Jolley et al. (1982) exposed islets of Langerhans from rabbits to low-frequency pulsed magnetic fields and noted a reduction in insulin release during glucose stimulation compared with controls (*p* < 0.002). Similarly, Navakatikyan et al. (1994) exposed rats to 50-Hz magnetic fields for 23 h per day for 11 days at 10, 50, and 250 μT. Serum insulin levels decreased at the middle- and high-flux densities, which the authors associated with stress.

Sakurai et al. (2004) measured insulin secretion from an islet derived insulinoma cell line, RIN-m, exposed to low-frequency magnetic fields of 5 mT compared with sham exposure of less than 0.5 μT. Insulin secretion was reduced by approximately 30% when exposed to low-frequency magnetic fields compared to sham exposure. The authors conclude: “it might be desirable for diabetic patients who have insufficient insulin secretion from pancreatic islets to avoid exposure to ELFMF”. The magnetic flux density was exceptionally high in this experiment and is unlikely to be encountered in normal daily life. Studies of the incipient level of electromagnetic exposure, at which insulin secretion is reduced, would be useful.

Li et al. (2005) exposed hepatocytes *in vitro* to 50 Hz pulsed electric fields (0.7 V/m) and noted a conformation change in the insulin molecule and an 87% reduction in the binding capacity of insulin to its receptors compared with controls.

Stress often increases plasma glucose levels in diabetics (Hinkle and Wolf, 1950; Jolley et al., 1982). Studies with laboratory animals and *in vitro* studies with human cells show both low-frequency electromagnetic fields and non thermal RF radiation stimulates production of stress proteins, and that the biochemical reactions are the same over a range of frequencies and intensities (Blank and Goodman, 2004). Release of insulin is strongly inhibited by the stress hormone norepinephrine, which leads to increased blood glucose levels during stress. Rajendra et al. (2004) found elevated levels of norepinephrine in the brain of fertilized chick eggs on day 15 following exposure to 5, 50, and 100 μT. The “stress response” to electromagnetic energy may provide, yet, another mechanism that could explain Type 3 diabetes.

Reduced insulin secretion and reduced binding capacity of insulin to its receptors may explain the elevated levels of plasma glucose in Type 3 diabetics exposed to electromagnetic fields. More research on mechanisms is needed.

## Conclusions

In addition to lifestyle and genetics, the environment appears to be another factor contributing to high levels of blood sugar. This concept presents a possible paradigm shift in the way we think about diabetes and the consequences may be far reaching. As a result, we have labeled environmental diabetes as Type 3 diabetes.

We recognize that there is, as yet, no accepted definition of Type 3 diabetes and that our definition may be in conflict with others that have been suggested including a combination of Type 1 and Type 2, gestational diabetes, and that Alzheimer’s Disease is a form of diabetes (Steen et al., 2005; de la Monte et al., 2006).

What we describe here is a totally different type in the sense it has an environmental trigger. Doctors have long suspected an environmental component but it has not been until now that one has been found.

The increasing exposure and ubiquitous nature of electromagnetic pollution may be contributing to the increasing incidence of this disease and the escalating cost of medical care. Diagnosis of diabetes needs to be done in an electromagnetically clean environment to prevent misdiagnosis, and to properly assess the severity of this disorder. Most medical centers have electronic equipment and use fluorescent lights that produce dirty electricity, which is likely to cause abnormally high blood sugar readings for those with a combination of diabetes and electrohypersensitivity (Type 3 diabetes). Dirty electricity may also explain why brittle diabetics have difficulty controlling their blood sugar levels.

Type 3 diabetes, as described in this study, is an emerging disease. Unlike true Type 1 and Type 2 diabetics whose blood sugar is not affected by dirty electricity, Type 3 diabetics may be better able to regulate their blood sugar with less medication, and those diagnosed as borderline or pre-diabetic may remain non diabetic longer by reducing their exposure to electromagnetic energy. The GS filters and the microsurge meter provide the tools needed for scientific investigation of dirty electricity and may help diabetics regulate their blood sugar by improving power quality in their home, school, and work environment. Minimizing exposure to radio frequencies (kHz to GHz), flowing along the ground or through the air, also needs to be addressed. Large-scale studies are needed in controlled settings to determine the percentage of the population with Type 3 diabetes.

These results are dramatic and warrant further investigation. If they are representative of what is happening worldwide, then electromagnetic pollution is adversely affecting the lives of millions of people.

## Footnotes

* Multiply by 18 to convert to mg/dL.

1 Both the short-acting Humalog® (H-insulin) and the long-lasting Humulin^s^ NPH (N-insulin) are produced by Eli Lilly.
